# Excluding embryos with two novel mutations in *FREM2* gene by the next-generation sequencing-based single nucleotide polymorphism haplotyping

**DOI:** 10.18632/aging.203715

**Published:** 2021-11-27

**Authors:** Yao Zhou, Xiaohui Yang, Zheng Liu, Yu Zhang, Huaye Chen, Yongfang Zhang, Yuxin Hu, Yanlin Ma, Qi Li

**Affiliations:** 1 Hainan Provincial Key Laboratory for Human Reproductive Medicine and Genetic Research, Reproductive Medical Center, The First Affiliated Hospital of Hainan Medical University, Hainan Medical University, Haikou 570102, Hainan, China; 2 Key Laboratory of Tropical Translational Medicine of Ministry of Education, Hainan Medical University, Haikou 571199, Hainan, China; 3 Hainan Provincial Clinical Research Center for Thalassemia, The First Affiliated Hospital of Hainan Medical University, Hainan Medical University, Haikou 570102, Hainan, China; 4 College of Medical Laboratory Science, Guilin Medical University, Guilin 541004, Guangxi, China; 5 Division of Biological Sciences, University of California San Diego, San Diego, CA 92093, USA

**Keywords:** Fraser syndrome, FREM2 gene, the next-generation sequencing-based single nucleotide polymorphism haplotyping, embryos selection

## Abstract

Fraser syndrome is a rare autosomal recessive malformation disorder. It is characterized by cryptophthalmos, syndactyly, urinary tract abnormalities and ambiguous genitalia. This condition is due to homozygous or heterozygous mutations in the *FRAS1*, *FREM1*, *FREM2*, and *GRIP1* genes*.* In the present study, we recruited a Chinese family with Fraser syndrome. Two novel mutations c.7542_7543insG and c.2689C>T in the *FREM2* gene were detected in this Fraser syndrome family by PCR-based sequencing. The next-generation sequencing-based single nucleotide polymorphism haplotyping method was applied to exclude these two mutations in 9 blastocysts obtained from the patient. After obtaining consent and informing the risk, the patient received *in vitro* fertilization and embryo transfer treatment with an embryo carrying a heterozygous mutation. Finally, she delivered a healthy baby without any complications on March 17, 2019. In conclusion, we first reported two novel mutations in the *FREM2* gene associated with the risk of Fraser syndrome. Moreover, we described a next-generation sequencing-based single nucleotide polymorphism haplotyping method to select the ‘right’ embryos from patients with Fraser syndrome for *in vitro* fertilization and embryo transfer treatment.

## INTRODUCTION

Fraser syndrome (FS) (MIM#219000) is a rare genetic disorder that often presents with ocular, renal, genital and limb's congenital anomalies [1]. The diagnosis of FS is based on major criteria (syndactyly, cryptophthalmos spectrum, urinary tract abnormalities, ambiguous genitalia, laryngeal and tracheal anomalies, positive family history) and minor criteria (anorectal defects, dysplastic ears, nasal anomalies, skull ossification defects, umbilical abnormalities) [2, 3]. In human, homozygous or heterozygous mutations in FS protein 1 (*FRAS1*), FRAS1-related extracellular matrix protein 1 (*FREM1*), *FREM2*, and glutamate receptor-interacting protein 1 (*GRIPI*) genes result in classic FS phenotype [4]. Functionally, *FRAS1*, *FREM1* and *FREM2* genes encode members of extracellular matrix proteins [5]. These proteins are secreted by mesenchymal cells during diaphragmatic development and form a ternary complex at the basement membrane, which plays a crucial role in forming human and rodent diaphragms [6]. *GRIP1* encodes a multi-PDZ domain-containing protein involved in the basolateral trafficking and export of FRAS1 and FREM2 in epithelial cells [7]. Defects of any of these proteins destabilize the extracellular matrix, causing dermal-epidermal detachment [8].

Clinically, the malformations of FS are challenging to detect prenatally [9]. In order to examine genetic disease or chromosome abnormalities in embryos before implantation to the uterus, as one of the most effective technologies, next-generation sequencing (NGS) in preimplantation genetic diagnosis (PGD) is developed [10]. It allows achieving normal pregnancy by transferring embryos without the target gene mutation. However, embryologists still face severe challenges in detecting mutations in human embryos due to a small number of embryos producing the issues of high amplification bias, low accuracy and reproducibility in NGS–based preimplantation genetic diagnosis [11]. Another challenge is from allele drop out (ADO) arising from non-amplification of one allele, which produces the false-negative result and typically results in misdiagnosis [12]. In recent years, as an economical, user-friendly, and accurate method, next-generation sequencing (NGS)-based single nucleotide polymorphism (SNP) haplotyping has been applied to detect the mutation in human preimplantation embryos [13, 14].

In this study, we first report two novel causative mutations in the *FREM2* gene in an FS family. Furthermore, NGS-based SNP haplotyping was used to help in selecting the ‘right’ embryos for *in vitro* fertilization treatment. Finally, the woman successfully conceived on this embryo transfer.

## RESULTS

### Two novel mutations in *FREM2*

**Figure 1:**
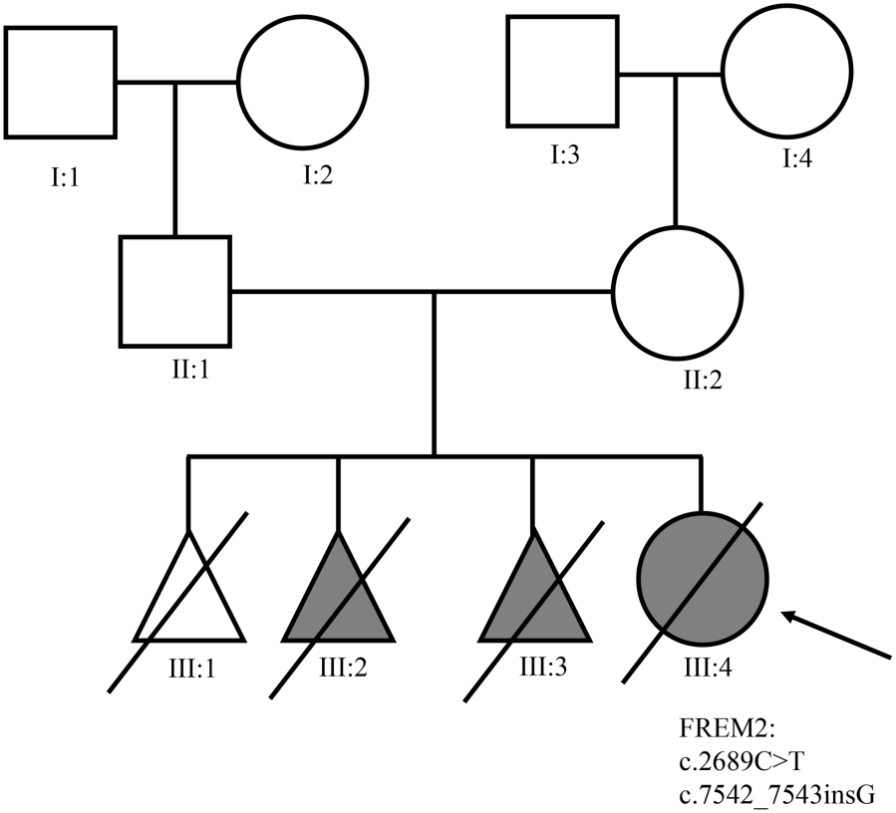
**The pedigree of the family with Fraser syndrome.** Roman numerals indicate generations, and individuals within a generation are numbered from left to right. The proband (III: 4) is denoted with an arrow.

**Figure 2:**
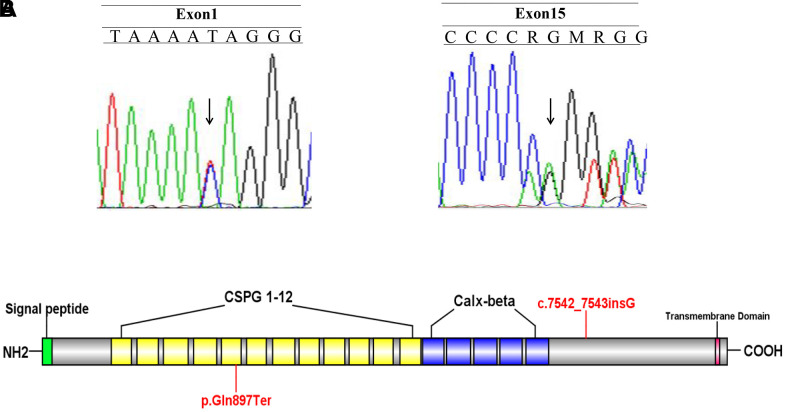
**Identify the disease-causing mutation in the Fraser syndrome family.** (**A**) Two mutations c.7542_7543insG and c.2689C>T were identified. (**B**) A schematic of the FREM2 protein and location of the mutations.

**Figure 3:**
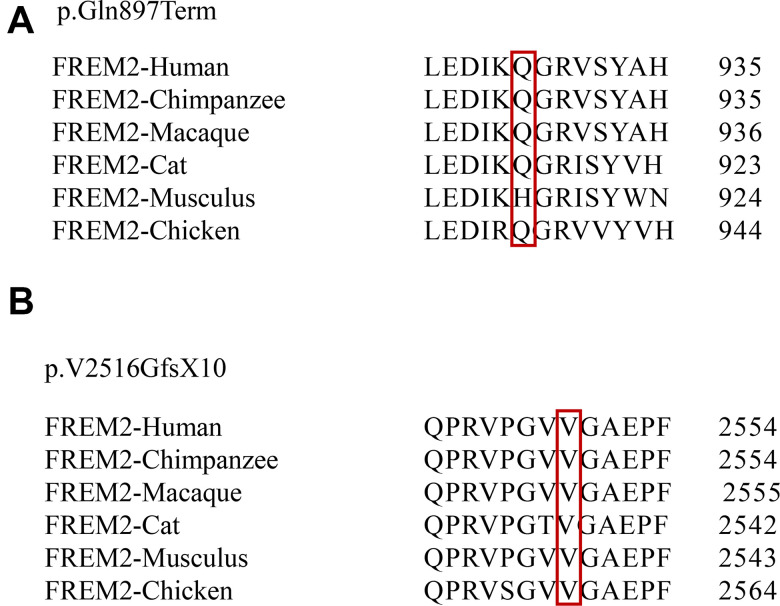
**Conservation analysis of affected amino acids among six primate species.** Evolutionary conservation of the mutations within FREM2 across species is analyzed. The positions of 2 mutations p.Gln897Term (**A**) and p.V2516GfsX10 (**B**) are indicated in red boxes.

A Chinese family with FS was recruited in this study (Figure 1). Two novel *FREM2* gene mutations were found: one frameshift mutation c.7542_7543insG was carried by the female (II:2), and another nonsense mutation c.2689C>T was carried by the male (II:1) (Figure 2A). These two mutations were not detected in 80 unrelated healthy individuals. The couple was introduced to our center for PGD testing. These two mutations in *FREM2* (c.7542_7543insG, p.V2516GfsX10; c.2689C>T, p.Gln897Ter) (Figure 2B) were submitted to the ClinVar database (http://www.ncbi.nlm.nih.gov/clinvar), and the received the IDs SUB5692939 and SUB5690409, respectively. Clustal analysis indicated that these two amino acids are highly conserved in FREM2 proteins across vertebrate species, including the chimpanzee, Macaque, Cat, Musculus, and Chicken (Figure 3).

Sanger sequencing revealed that the mutation c.2689C>T (p.Gln897Ter) in the male (II:1) was inherited from his father I:1. The mutation c.7542_7543insG (p.V2516GfsX10) in female II:2 was inherited from her mother I:4 (Table 1). These two mutations were identified at the level of PVS1 according to the guidelines for assigning disease causality. Mutation Taster and PROVEAN software predicted both mutations appeared to produce truncated proteins, which seriously affected the function of FREM2.

**Table 1:** Sequencing results of the family members.

	**c.2689C>T, p.Gln897Ter**	**c.7542_7543insG, p.V2516GfsX10**
Female	Wild-type	Heterozygous mutation
Male	Heterozygous mutation	Wild-type
Female’ father	Wild-type	Wild-type
Female’ mother	Wild-type	Heterozygous mutation
Male’ father	Heterozygous mutation	Wild-type
Male’ mother	Wild-type	Wild-type
Fetus	Heterozygous mutation	Wild-type

### Biopsied trophectoderm cells

In order to help these couples with the issues of *FREM2* mutations in having a healthy baby, seventeen matured oocytes from the patients at MII stage were collected after ovarian stimulation and then fertilized by intracytoplasmic sperm injection. Nine blastocysts were utilized for trophectoderm biopsy when the trophectoderm cells herniated out of the zona pellucid on day 5 or 6 after fertilization.

### Pedigree haplotype construction and linkage analyses

Analysis of the detected SNP loci suggested that 68 SNP loci, including 20 loci from the female (II:2) and 48 loci from the male (II:1), could provide information for linkage analyses. Among these 68 loci, 5 were on the FREM2 gene, including 4 in the female (II:2) and 1 in the male (II:1). The pedigree haplotype and linkage analysis were used to determine whether the embryo had carried the mutations. For example, on SNP rs38089800, the male had homozygous C/C, and the female had heterozygous T/C, suggesting that the male transmitted “C” and the female transmitted “T” to E1. Meanwhile, the linkage analysis showed that E5 and E15 were wild-types, E4, E7, E8 and E14 were heterozygous carriers, and E1, E2 and E17 were homozygous carriers (Table 2).

**Table 2:** Twenty-two SNP markers were respectively selected for identifying disease-associated allele in the embryos.

**ID**	**Chr**	**POS**	**REF**	**ALT**	**MF**	**MM**	**M**	**FF**	**FM**	**F**	**E1**	**E2**	**E4**	**E5**	**E7**	**E8**	**E14**	**E15**	**E17**
FREM2_8	Chr13	38089800	C	T	C/T	C/C	C/C	C/C	T/T	T/C	C/T	C/T	C/T	C/C	C/C	C/C	C/T	C/T	C/T
FREM2_9	Chr13	38174781	T	C	T/C	T/T	T/T	T/T	C/C	C/T	T/C	T//C	T/T	T/T	T/T	T/T	T/T	?/?	T/C
FREM2_14	Chr13	38361871	A	G	G/G	G/G	G/G	G/A	G/A	G/A	G/G	G/G	G/G	G/A	G/A	G/A	G/G	G/A	G/G
FREM2_F_2	Chr13	38382809	A	G	A/A	G/G	A/G	G/A	A/A	A/A	G/G	G/G	G/A	G/A	A/A	G/A	G/A	G/A	A/A
FREM2_15	Chr13	38382875	C	T	C/C	T/T	C/T	T/C	C/C	C/C	C/C	C/C	T/C	T/C	C/C	T/C	T/C	T/C	C/C
FREM2_F_3	Chr13	38382982	G	C	G/G	C/C	G/C	C/G	G/G	G/G	G/G	G/G	C/G	C/G	G/G	C/G	C/G	C/G	G/G
FREM2_16	Chr13	38406297	G	A	G/G	A/A	G/A	A/G	G/G	G/G	G/G	G/G	A/G	A/G	G/G	A/G	A/G	A/G	G/G
FREM2_F_4	Chr13	38406309	C	A	C/C	A/A	C/A	A/C	C/C	C/C	C/C	C/C	A/C	A/C	C/C	A/C	A/C	A/C	C/C
FREM2_F_6	Chr13	38501932	G	T	G/G	T/T	G/T	G/G	G/G	G/G	G/G	G/G	T/G	T/G	G/G	T/G	T/G	T/G	G/G
FREM2_22	Chr13	38552205	G	A	G/G	A/A	G/A	G/G	G/G	G/G	G/G	G/G	A/G	A/G	G/G	A/G	A/G	A/G	G/G
FREM2_M_1	Chr13	38625874	G	A	G/G	G/G	G/G	G/A	G/G	G/A	G/G	G/G	G/G	G/A	G/A	G/A	G/G	?/?	G/G
FREM2_F_14	Chr13	39264690	T	C	T/T	T/C	T/C	T/T	T/T	T/T	??	??	C/T	C/T	?/?	?/?	C/T	?/?	?/?
FREM2_M_7	Chr13	39430443	G	A	G/G	G/G	G/G	A/A	G/G	G/A	G/G	G/G	G/G	G/A	G/A	G/G	G/G	G/A	G/G
FREM2_M_8	Chr13	39431945	T	C	T/T	T/T	T/T	C/C	T/T	T/C	T/T	??	T/T	??	?/?	T/T	T/T	?/?	?/?
FREM2_M_10	Chr13	39446869	G	A	G/G	G/G	G/G	A/A	G/G	G/A	G/G	??	G/G	G/A	G/A	G/G	G/G	G/A	?/?
FREM2_M_11	Chr13	39452934	C	T	C/T	T/C	C/C	C/C	T/T	T/C	??	C/T	C/T	C/C	C/C	?/?	C/T	C/C	C/T
FREM2_62	Chr13	39477115	G	C	C/G	C/C	C/C	C/C	G/G	G/C	C/G	C/G	C/G	C/C	C/C	C/G	C/G	C/C	C/G
FREM2_80	Chr13	39792426	C	T	C/C	T/C	C/C	T/T	C/C	C/T	C/C	C/C	C/C	T/C	C/T	C/C	C/C	C/T	C/C
FREM2_82	Chr13	39821473	G	A	G/G	A/G	G/G	G/A	G/G	G/A	G/G	G/G	G/G	A/G	G/A	G/G	G/G	G/A	G/G
FREM2_90	Chr13	39949981	T	C	T/T	C/C	T/C	T/T	T/T	T/T	T/T	T/T	C/T	T/C	T/T	C/T	C/T	C/T	T/T
FREM2_M_12	Chr13	39991091	T	A	T/T	A/T	T/T	T/T	A/T	A/T	T/A	T/A	T/A	T/T	T/T	T/A	T/A	T/T	T/A
FREM2_111	Chr13	40464364	G	A	G/A	A/A	G/A	A/A	A/A	A/A	G/A	G/A	A/A	A/A	G/A	A/A	A/A	A/A	G/A

E5 and E15 were wild-type, E4, E7, E8 and E14 were heterozygous carriers, and E1, E2 and E17 were carried two pathogenic mutations. ID, reference SNP cluster ID; Chr, chromosome number; POS, genomic position; REF, reference allele of the SNPs; ALT, alternative allele of the SNPs; FF, female’s father; FM, female’s mother; MF, male’s father; MM, male’s mother; E, embryo. The red base indicates associated mutation, while the black base indicates that the SNP links with wild type. The green background indicates uncertain recombination. “?”means that the alleles are not covered.

### Clinical outcomes

Wild-type blastocysts E5 and E15 were selected for the first two transplantations. Unfortunately, no clinical pregnancy was achieved. We then transferred the heterozygous blastocyst E4 with a good grade, which still did not generate a clinical pregnancy. The first three transplantations were all carried out under the artificial cycle scheme. Due to repeated implantation failure, the woman was recommended to perform hysteroscopy. It showed that the uterine cavity had adhesion on the right side. The shape of the uterine cavity returned to normal after operation. Endometrial tissue pathology suggested that the proliferative endometrium was associated with polyp formation.

**Figure 4:**
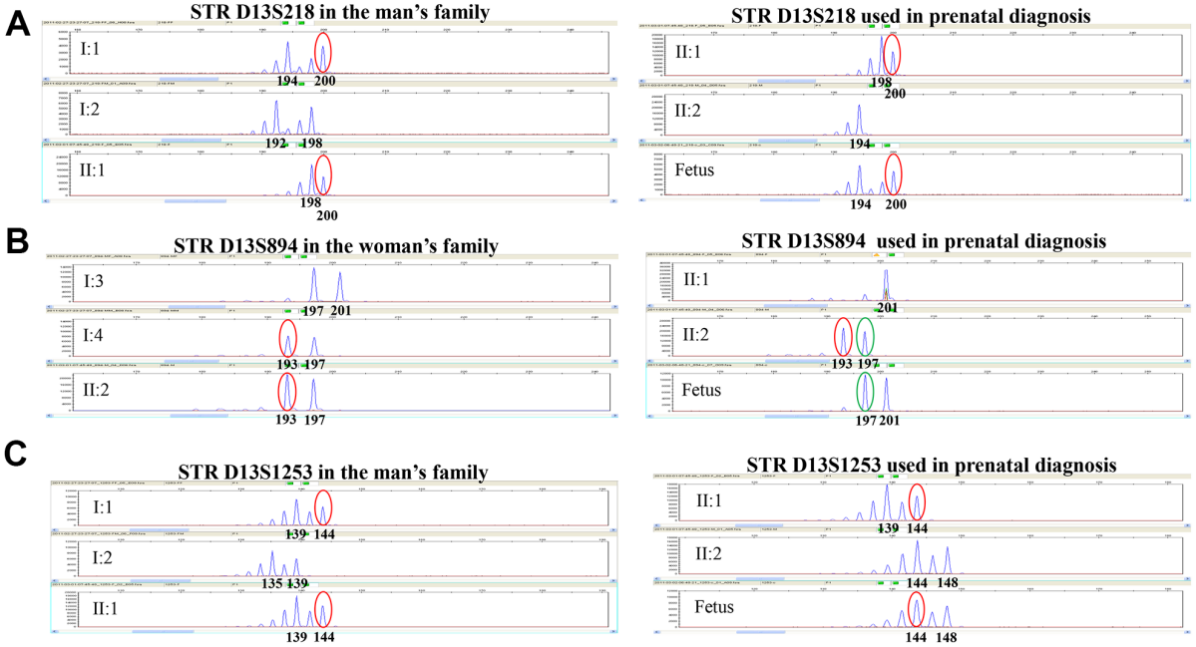
**Genotyping results of STR D13S218, STR D13S894, and STR D13S1253 in this family.** STR D13S218, D13S894 and D13S1253 showed that the fetus was a heterozygous carrier with one *FREM2* gene mutation (c.2689C>T, p.Gln897Ter) inherited from the father, which is consistent with the PGD result. (**A**) Genotyping for STR D13S218 in the man’s family and used in prenatal diagnosis indicates that the chromosome carrying pathogenic mutation originated from his father. The fetus inherited pathogenic chromosome from his father. (**B**) Genotyping for STR D13S894 in the woman’s family and used in prenatal diagnosis indicates that the chromosome carrying pathogenic mutation originated from her mother. The fetus inherited the normal chromosome from his mother. (**C**) Genotyping for STR D13S1253 in the man’s family and used in prenatal diagnosis indicates that the chromosome carrying pathogenic mutation originated from his father. The fetus inherited the pathogenic chromosome from his father.

We then adopted the down-regulation scheme combined with the artificial cycle scheme to transplant E7 blastocyst. The woman finally got a clinical pregnancy. Sequencing of the fetal amniotic cell’s DNA revealed that the fetus was a heterozygous carrier with one *FREM2* gene mutation (c.2689C>T, p.Gln897Ter). The mutation was inherited from its father (II1). The fetus’ karyotype was normal. The linkage analysis, D13S218, D13S894 and D13s1253 STR markers confirmed the above findings (Figure 4). Thus, continued pregnancy was recommended. During pregnancy, all ultrasonographic examinations did not show any morphological fetal abnormality. The baby was born on March 17, 2019 and showed normal development and growth after a follow-up of 12 months.

## DISCUSSION

FS is a rare genetic malformation with an autosomal recessive inheritance pattern in which the life expectancy is <1 year [15]. Genetics factors are well recognized in the cause of FS, but the underlying causes of FS remain unclear [16]. In the present study, the woman (II: 2) terminated pregnancies for fetal abnormality. PCR-based sequencing revealed that the woman carried a novel mutation c.7542_7543insG, and her husband (II: 1) carried a novel mutation c.2689C>T. Further analysis revealed that the woman’s mutation inherited from her mother (I: 4) and her husband’s mutation inherited from his father (I: 1). Since the postmortem examination of the proband (III: 4) conformed to the clinical diagnostic criteria of FS, we analyzed the pathogenicity of these two novel mutations in the *FREM2* gene. The mutation p.Gln897Ter is located in the fifth chondroitin sulfate proteoglycan (CSPG) domain and can produce a truncated protein. The mutation p.V2516GfsX10 is closely located with the Calx-β domain and also produce a truncated protein. Many experiments demonstrated that a stop codon resulting in a truncated protein is considered to lose the protein biological function [17]. The possible pathogenic mechanism of FS patients with p.Gln897Ter mutation is that this mutation produces a truncated protein with a break CSPG domain and without Calx-β domain. Both of these two truncated proteins will lose the transmembrane domain of FREM2 protein. Therefore, we here predicted both nonsense mutations have strong evidence in favor of disease-causing potential.

To date, 45 disease-causing mutations, including 6 nonsense mutation in the FREM2 gene, have been reported in HGMD Professional (https://portal.biobase-international.com/hgmd/pro/all.php). However, only four mutations including a substitution c. 5914G>A in the second of 5 consecutive Calx-β domains, a splicing mutation IVS14+1G >A resulting in a stop codon at residue 2549, a substitution mutation c.6499C>T, and a deletion mutation c.15delG in FREM2 gene have been reported to be associated with a risk of FS in OMIM database [18, 19]. In order to exclude the embryo containing no suspected mutations, PGD was introduced in the late 1980s and allows the detection of FREM2 gene mutations in embryos produced through *in vitro* fertilization [20]. Today, advanced molecular technologies with better resolution, such as array comparative genomic hybridization, quantitative PCR, and NGS, are on the verge of becoming the gold standard in embryo preimplantation screening [21, 22]. However, amplification failure and ADO are two common problems caused by NGS sequencing errors [23, 24]. In the present study, we describe the methods based on NGS-based SNP haplotype to distinguish the wild-type, heterozygous carriers, and homozygous carriers of FS embryos. Our results revealed that the D13S218, D13S894 and D13s1253 STR markers could be used to exclude the mutations c.7542_7543insG and c.2689C>T in FS patients. For this couple, the wild-type embryos and heterozygous carriers could be used to transfer, but the risk should be informed. The end of this story is that they accept to undergo embryo transfer and obtained one healthy baby who is a heterozygous carrier.

In conclusion, in the present study, we report two novel mutations in *FREM2* gene associated with the risk of FS. Next, we describe a method based on NGS-based SNP haplotype to exclude the embryos carried homozygous mutation. Lastly, the patients received an embryo with heterozygous mutation and obtained one healthy baby.

## MATERIALS AND METHODS

### Ethics statement

This study followed the principles of the Declaration of Helsinki. Informed consent was obtained from all participants. The study was approved by the institutional ethics committee of The First Affiliated Hospital of Hainan Medical University.

### Family history

II: 1 (male, 32 years old) and II: 2 (female, 31 years old) had no clinical symptoms of FS (Figure 1). The female had pregnancies four times, including three adverse pregnancies (III:1, III:2, and III:3). The last pregnancy was terminated at 27 weeks of gestation. Prenatal ultrasound examination revealed that the proband (III:4) had 4.0 cm thickness of fetal ascites, edema of fetal skin, and a large amount of fluid in fetal abdominal cavity and enlargement of lungs. Postmortem examination showed that the proband did not have a left eyeball. It had bilateral syndactyly (toe) and mal-developed right kidney, which conformed to the three major diagnostic criteria of FS. Also, the location of both ears was symmetrically low, and the umbilical cord was swollen, which were the two minor criteria for FS diagnosis.

### Mutation screening

The DNA samples from the aborted proband (III:4) and the couple (II:1 and II:2) were screened by next-generation sequencing in Beijing Genomics Institution. We confirmed the mutations in *FREM2* gene by Sanger sequencing. The PCR primers are shown in Table 3. The pathogenicity of all the mutations were evaluated according to the guidelines of the American College of Medical Genetics (ACMG), Mutation Taster and Provean software. All sequencing readouts were mapped to compare to the *FREM2* reference genome on NCBI (NM_207361.6). Eighty human random control DNAs were set as healthy control.

**Table 3:** Primers used in this study.

**Mutations**	**Primers**	**Sequence(5’-3’)**	**Genomic location**
c.2689C**>**T	FREM2_1F	GTCCTCAACACCGGCTTCA	chr13:39261173-39461268
	FREM2_1R	GAGTGCCAGTAGGATGGCTC	
c.7542_7543insG	FREM2_15F	TCCACAGAGAAGTTGAAAGTACACA	
	FREM2_15R	GCTTACCCAAGTCACCTACCA	
D13S218	D13S218F	GATTTGAAAATGAGCAGTCC	chr13:38458094-38458429
	D13S218R	GTCGGGCACTACGTTTATCT	
D13S1288	D13S1288F	TTCAGAGACCATCACGGC	chr13:38949133-38949542
	D13S1288R	CTGGAAAAATCAGTTGAATCCTAGC	
D13S1253	D13S1253F	CCTGCATTTGTGTACGTGT	chr13:39572161-39572531
	D13S1253R	CAGAGCCGTGGTAGTATATTTTT	

### Whole-genome amplification

Biopsied trophectoderm cells were washed in PBS (with 0.1% HAS) and transferred to the PCR tube. The whole genome of the cell was amplified by the Pico PLEX single-cell WGA kit (NEB-WGA) according to the manufacturer’s protocol. The sequencing library was constructed by a personalized genome library-building Kit (zykw-c-003) produced by Peking Jabrehoo Med Tech Co. Ltd. Sequencing was performed with the Ion Torrent Personal Genome Machine (Life technology, USA). At the same time, WGA products were also re-amplified using primers surrounding the mutated site, as shown in Table 3.

### Haplotype construction

Since the DNA sample of the proband (III:4) was missing, haplotype construction was generated from the couple (II:1, II:2) and their parents (I:1, I:2, I:3 and I:4). A total of 120 linked SNPs markers located in 5’ and 3’ regions of *FREM2* were analyzed. The genomic DNA of this pedigree was sequenced by NGS. The SNP readouts at the adjacent positions allowed the pathogenic alleles to be identified. A similar strategy was used to determine whether each embryos’ pathogenic allele was present from the informative SNPs in embryos.

### Embryo transfer

Blastocysts with normal or heterozygous mutations without chromosomal abnormality were selected for transplantation. Only 1 blastocyst was transplanted per resuscitation cycle. At 17+ weeks of gestation, 20 mL of the fetal amniotic fluid is extracted for DNA extraction, cell culture, and subsequent prenatal diagnosis.

### Prenatal diagnosis

The *FREM2* gene mutation was detected by Sanger sequencing. For prenatal diagnosis and linkage analysis, 4 STR markers which are located in the regions of the *FREM2* gene were chosen, with each STR’s forward primer being modified with Fam fluorescence. These primer sequences are shown in Table 3. After PCR amplification, the products were examined by fluorescence electrophoresis by ABI-3500 (Applied Biosystems, Foster City, CA, USA). A detailed prenatal ultrasound examination was performed to eliminate the chance of fetal malformations after a successful pregnancy.
